# Effect of swimming intensity on performance in subsequent sprint triathlon: a sex-based analysis in amateur athletes

**DOI:** 10.1007/s00421-025-06062-z

**Published:** 2025-12-04

**Authors:** Lavínia Vivan, Vinícius Ribeiro dos Anjos Souza, Paulo Engelke, Claudio Andre Barbosa de Lira, Rodrigo Luiz Vancini, Katja Weiss, Beat Knechtle, Marilia Santos Andrade

**Affiliations:** 1 Postgraduate Program in Translation Medicine, Federal University of São Paulo, São Paulo, Brazil; 2 Human and Exercise Physiology Division, Faculty of Physical Education and Dance, Federal University of Goiás, Goiânia, Brazil; 3 Center for Physical Education and Sports, Federal University of Espírito Santo, Vitória, Brazil; 4 Institute of Primary Care, University of Zurich, Zurich, Switzerland; 5 Medbase St. Gallen Am Vadianplatz, St. Gallen, Switzerland; 6 Department of Physiology, Federal University of São Paulo, São Paulo, Brazil

**Keywords:** Amateur athletes, Sports, Sex differences, Performance analysis, Triathlon

## Abstract

**Purpose:**

Generally, triathletes participating in short events should aim to finish the swim stage with the leading group to improve their chances in the subsequent cycling and running stages. However, the fatigue associated with very intense swimming can impair subsequent split times. This study aimed to investigate the impact of swimming bout intensity on performance in subsequent cycling, running, and overall racing among well-trained male and female amateur triathletes.

**Methods:**

Twenty athletes (12 men and 8 women) participated in this study. Critical velocity (CV) was estimated for swimming using a simple linear model. Body composition and maximal oxygen uptake were measured. Participants visited the laboratory three times to swim 750 m at intensities below CV, at CV, and above CV (in randomized order), before they cycled 20 km and ran 5 km as fast as they could to simulate a sprint triathlon. Heart rate, blood lactate level, perceived exertion, muscle pain, and dyspnea levels were measured at the end of each modality.

**Results:**

For women, on the day on which swimming intensity was below the CV, the overall race time was higher than on the day on which swimming intensity was at the CV (*p* = 0.041) or faster than the CV (*p* = 0.002). For men, there was no significant difference in the overall race time among the three intensities (*p* > 0.05).

**Conclusion:**

The results showed that, for men, swimming at higher intensity did not significantly change total time, whereas for women, lower intensity was associated with poorer performance.

**Clinical trial number:**

The study was registered in ReBEC - Registro Brasileiro de Ensaios Clinicos: Rio de Janeiro (RJ): Instituto de Informação Científica e Tecnológica em Saúde (Brazil); 2010 - Identifier RBR-73vcyff. Available from http://ensaiosclinicos.gov.br/rg/RBR-73vcyff.

## Introduction

Triathlon is an endurance sport that comprises sequential swimming, cycling, and running (Etxebarria et al. 2019). Its main categories are the sprint distance (0.75 km swimming, 20 km cycling, and 5 km running), Olympic distance (1.5 km swimming, 40 km cycling, and 10 km running), Half IRONMAN^®^ distance (IM 70.3; 1.9 km swimming, 90 km cycling, and 21 km running), and full distance IRONMAN^®^ (IM 140.6; 3.8 km swimming, 180 km cycling, and 42 km running) (Sousa et al. 2021). The number of shorter races has been growing recently, possibly because of the Super League Triathlon series (variable short-course distances) and the inclusion of the mixed relay event at the 2020 Tokyo Olympics (Walsh 2019).

In triathlon, the three disciplines do not contribute equally to the total time. The contribution of each discipline to overall performance varies significantly across the various triathlon distances, such as sprint, Olympic, and IRONMAN^®^ (Figueiredo et al. 2016). For example, the proportion of time spent swimming in Sprint or Olympic distance events is approximately 17%, while it is around 10% in IRONMAN^®^ 70.3 or IRONMAN^®^ 140.6 events (Sousa et al. 2021).

The last two stages of the race (cycling and running) are crucial in determining an athlete’s final position in the overall race, as they have the most significant contribution to overall performance (Figueiredo et al. 2016). In short-distance events, swimming performance is particularly decisive, since exiting the water with the lead pack greatly increases the likelihood of sustaining a competitive position during the cycling leg and ultimately securing a strong final result (Landers et al. 2008; Vleck et al. 2008). However, the very intense swimming segment imposes substantial physiological stress, including elevations in heart rate, blood lactate, and core temperature, which collectively impair cardiovascular stability and increase rating of perceived exertion. These alterations can reduce gross efficiency and compromise power output during the subsequent cycling phase, particularly in short-distance triathlon events (Ambrosini et al. 2022). Although the use of a wetsuit or swimming in a drafting position may attenuate these deleterious effects, the swim-to-cycle transition remains a critical determinant of reduced cycling performance (Wu et al. 2014; Puce et al. 2022). In the same direction, comparisons between isolated running and the running segment performed after swimming and cycling consistently demonstrate a reduction in running performance during triathlon events (Takahashi and Nabekura 2021). Therefore, it is necessary to develop a well-planned strategy throughout the race to optimize the intensity of effort during swimming to avoid fatigue and achieve satisfactory performance in the cycling and running stages (Puccinelli et al. 2020; Barbosa et al. 2023).

A possible method for measuring performance and planning swimming training intensity is using critical velocity (CV) as a reference (Toubekis et al. 2011a; Demarie et al. 2022; Petrigna et al. 2022). CV is the highest speed that can be sustained based on maximal aerobic power (di Prampero et al. 2008). It represents the maximum speed a swimmer can maintain over time without exhaustion (Campos et al. 2010). Nevertheless, the ideal CV percentage that an athlete should target for the swim stage of a sprint triathlon to guarantee the best performance without compromising cycling and running output remains unresolved. This should be examined separately for male and female athletes, as previous research has shown that pacing strategies during triathlons differ between sexes (Vleck et al. 2008). These differences are probably due to morphological, biomechanical, physiological, and metabolic differences, such as variations in fat body mass, muscle fiber type percentage, and carbohydrate metabolism (Tarnopolsky 2008).

For instance, it has been previously demonstrated that women are able to sustain a higher percentage of their respiratory compensation point during the running segment of a triathlon (De Araújo Moury Fernandes et al. 2023), likely due to a greater proportion of type I fibers, more efficient lipid utilization, and lower fatigability during prolonged exercise. These adaptations enable women to maintain higher relative intensities despite lower absolute VO₂max values and running velocities (Hunter 2014). Despite the remarkable differences between the sexes, exercise science has traditionally focused on male subjects (Besson et al. 2022), therefore, research involving female athletes is warranted and especially important for planning effective race strategies.

In light of these gaps, this study investigated the effect of swimming intensity on performance in subsequent cycling, running, and overall racing among well-trained male and female amateur triathletes. Another objective of the study was to compare internal (heart rate, lactate, perceived exertion, muscle pain, and dyspnea) and external (performance/time) load measures following each daily session of swimming, cycling, or running. This study hypothesizes that intense swimming may impair subsequent cycling and running performance, with potential consequences for the overall race outcome. Furthermore, we hypothesized that cycling and running are less impacted by swimming intensity in women than in men, given that women presented a greater fat oxidation compared with men. (Tarnopolsky 2008).

## Materials and methods

### Ethical approval

All experimental procedures were approved by the human research ethics committee of the relevant institution and adhered to the principles outlined in the Declaration of Helsinki. All study participants provided written informed consent. The study was approved by the Research Ethics Committee of the Federal University of São Paulo under number 7.351.985.

### Participants

Well-trained triathletes, engaging in six or more hours per week of swimming, cycling, and running, were recruited through social media, triathlon coaches, and sports consultancies. This minimum weekly training volume corresponded to the lowest amount of training reported among the athletes in a previous study (Clemente-Suárez et al. 2019). The inclusion criteria of the study were as follows: having at least 1 year of experience in triathlon and a medical approval to undergo a maximum effort test. Participants were excluded if they had any lower limb injury, were pregnant, or had a history of chronic disease. Initially, 12 men and 10 women were recruited; however, two women withdrew from the study due to their inability to complete all the laboratory visits. Therefore, a total of 20 athletes were included, comprising 12 men and 8 women. Descriptive data for the participants are presented in Table 1. In the male group, the weekly training load distribution comprised 157 ± 65 min of swimming, 284 ± 141 min of cycling, and 212 ± 66 min of running. In the female group, the weekly training load distribution comprised 157 ± 65 min of swimming, 284 ± 93 min of cycling, and 212 ± 59 min of running.

**Table 1:** Descriptive data for male and female triathletes

	Women (n = 8)	Men (n = 12)	*p* value	Cohen’s *d*	Power
Age (years)	38.38 ± 7.57	35.75 ± 11.01	0.565	0.267 (− 0.65 to 1.16)	0.77
Total body mass (kg)	59.5 ± 5.6	76.9 ± 6.3	< 0.001	− 2.887 (− 4.59 to − 1.14)	0.99
Height (cm)	162 ± 5	177± 4	< 0.001	− 3.204 (− 5.05 to − 5.32)	0.99
Lean mass (kg)	42.11 ± 4.59	60.24 ± 5.90	< 0.001	− 3.336 (− 5.24 to − 1.39)	0.99
% fat mass	24.5 ± 7.2	17.4 ± 6.4	0.034	1.049 (− 0.02 to 2.07)	0.64
BMD (g/cm^2^)	1.21 ± 0.09	1.34 ± 0.09	0.007	− 1.400 (− 2.52 to − 0.22)	0.64
Experience (years)	3.0 ± 1.2	3.3 ± 1.7	0.656	− 0.216 (− 1.15 to 0.73)	0.81
V̇̇O_2_ max (mL/kg/min)	48.40 ± 5.52	50.60 ± 5.44	0.399	− 0.394 (− 1.30 to 0.54)	0.73
CV (m/s)	0.81 ± 0.12	0.97 ± 0.11	0.009	− 1.333 (− 2.43 to − 0.18)	0.62

Data are presented as mean ± standard deviation. Confidence interval = 95%. V̇O_2_max, maximal oxygen uptake

*AT* Anaerobic threshold, *RCP* Respiratory compensation point, *MAP* Maximal aerobic power

### Study design

**Fig. 1:**
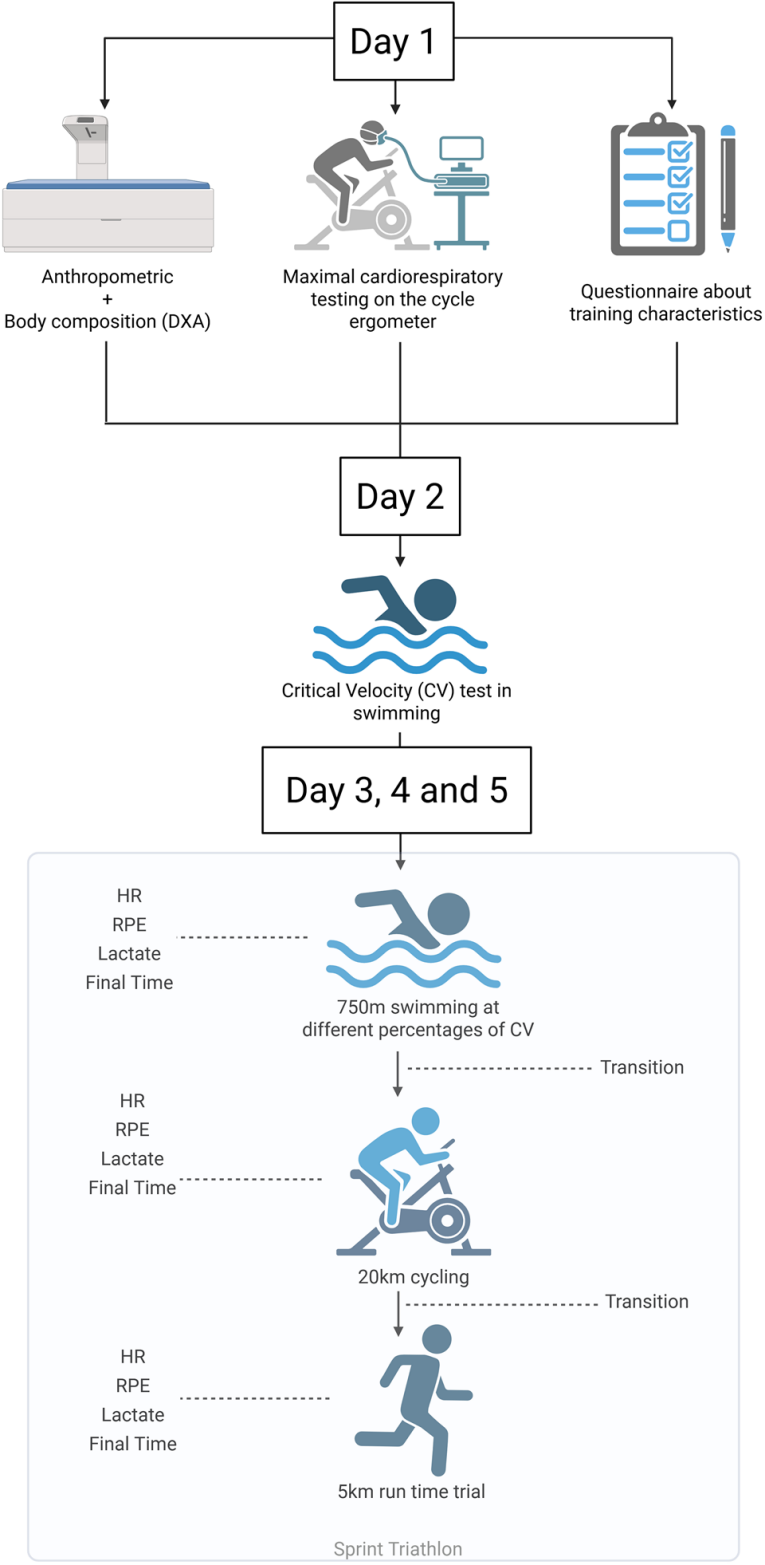
Experimental protocol of the study. Created in BioRender

The tests were performed during five visits to the Exercise Physiology Laboratory, according to Fig. 1. During the first visit, participants underwent anthropometric and body composition assessments, maximal exercise cardiorespiratory testing on a cycle ergometer, and completed a questionnaire on training characteristics. On the second visit, two swimming tests (200 and 400 m) were performed in a pool for each participant, and then CV was calculated. In the last three visits, the experimental protocol was implemented; it consisted of swimming 750 m in the pool at three intensities (slower than CV, at CV, faster than CV) in a random order using CV as a parameter, before cycling 20 km on a cycloergometer, and running a 5 km time trial on an athletics track, simulating a Triathlon sprint. An interval of 2–4 days was allowed between visits 1 and 2, and an interval of 7 days between visits 3, 4, and 5. The visits were conducted at the same time of day. There were no medical complications among the participants during experimental procedures.

## Experimental procedures

### Questionnaire

The questionnaire included the following open questions to understand training volume throughout the weeks of the protocol (Vivan et al. 2025). How long have you been training for triathlons? How many hours do you run per week? How many hours do you cycle per week? How many hours do you swim per week?

### Anthropometric and body composition tests

Dual-energy X-ray absorptiometry (DXA, GE Healthcare, USA) was used to determine the body composition (fat and fat-free mass) of the participants. DXA is a valid method, and a typical error of measurement for lower limb lean mass is 1.3% (Andreoli et al. 2009). The participants were instructed to wear comfortable clothes without metal pieces. During the test, each participant assumed a supine position with a 10-cm distance between the feet and 5 cm between the hands and trunk. All the tests were performed by the same experienced examiner. A calibrated stadiometer Filizola PL (Filizola, São Paulo, Brazil) was used to measure body mass and body height to the nearest 0.1 kg and 0.1 cm, respectively.

### Cardiorespiratory maximal cycle ergometer test

A cardiorespiratory exercise maximal test was performed on a cycle ergometer (Excalibur Sport 2019, Lode, Netherlands) using a computer and respiratory gas exchange analyzer (Quark, Cosmed, Italy) to determine the ventilatory threshold (VT), respiratory compensation point (RCP), maximum oxygen consumption (V̇O_2_max), and maximal aerobic power (MAP). The cardiorespiratory exercise maximal test was employed with the specific aim of characterizing the fitness profile of the study participants. The respiratory gas exchange analyzer was calibrated prior to each test according to the manufacturer’s guidelines and it presented good accuracy, with a consistently acceptable error (Van Hooren et al. 2024).

The test started with a 3-min warm-up at 85 and 130 W for women and men, respectively, followed by 15-W per min increases until volitional exhaustion. Different protocols were used for males and females, ensuring that the test lasted between 8 and 12 min in each case (Buchfuhrer et al. 1983). Respiratory data were collected breath by breath and averaged every 20 s for analysis. The Borg scale (Noble et al. 1983) was used to assess rating perceived exertion in each minute of the test. The determination of VT and RCP was based on Wasserman’s criteria. (Wasserman et al. 1973). VT and RCP were determined separately by two experienced investigators; a third investigator was asked in cases of discordance.

V̇O_2_ max was identified by a plateau in oxygen uptake at the end of the test. The plateau was defined as an increase in V̇O_2_ of less than 1.5 mL/kg/min even after a power increase (Buchfuhrer et al. 1983). In the absence of an apparent V̇O_2_ plateau, three criteria were required to obtain V̇O_2_max—a respiratory exchange ratio value of ≥1.15, a maximal heart rate value (HRmax) greater than 85% of the age-predicted maximum (207–0.7 × age) (Gellish et al. 2007), and exhaustion according to the Borg scale (Noble et al. 1983). The findings of this study showed that all of the participants reached V̇O_2_max. The minimal power associated with V̇O_2_max (MAP) was determined (Wasserman 1986).

### Critical velocity test

Protocols for determining CV in swimming involve a combination of performance trials and metabolic parameters. In this study, the protocol involved a randomized performance assessment of 200 and 400-m maximal front crawl bouts at a 25-m swimming pool. This protocol demonstrated high reliability, making it a viable option for swimmers (Costa et al. 2009; Toubekis et al. 2011b). The time to complete each distance was recorded in seconds using a manual stopwatch. The linear relationship model between time and distance was determined, and CV was determined as the slope of the regression line, according to Wakayoshi et al (Wakayoshi et al. 1992).

The CV test was used to determine swimming intensity. The protocol started with a warm-up for 10 min of easy intensity (self-select) in a 25-m long swimming pool. After a 5-min break, participants performed a 200-m time trial. After 10 min of rest, when heart rate (HR), rating perceived exertion, and dyspnea had returned to baseline values, participants performed a 400-m time trial. They were instructed to complete both distances in the shortest time possible.

### Sprint triathlon races

The warm-up procedure was the same as that for the CV test. The experimental test began after a 1-min recovery. The experimental test consisted of swimming 750 m in the pool at three different intensities (day 1–slower than CV, day 2–at CV, and day 3–faster than CV) in randomized order. Participants were blinded for swimming intensity and, they were instructed to maintain the pre-set velocity by following visual and verbal feedback provided by the investigators throughout the trials. Specifically, an experimenter walked alongside the pool, monitoring the swimmers’ pace with a digital chronograph and giving continuous feedback to ensure adherence to the prescribed velocity.

The target swimming speeds for the athletes were 3% lower than their CV on day 1 and, higher than their CV on day 3. The three-day swimming tests were performed in a 25-m long swimming pool.

After completing the 750 m of swimming, the participants changed to a cycling time trial (TT) of 20 km on an electronically braked cycle ergometer (Excalibur Sport, Lode, Netherlands). After finishing the 20-km cycling TT, the participants transitioned to 5-km running TT on the athletics track, simulating a sprint triathlon event. The first transition (T1) and the second transition (T2) were similar to those performed in a real race. At the end of each modality, blood lactate was collected from the earlobe using a lactate meter (Lactate Plus Meter Nova Biomedical, Waltham, MA, USA), HR was measured using a sensor attached to the cap (Polar Verity Sense, Polar Electro Oy, Finland), and the rating of perceived exertion (RPE) was measured by presenting the participants with the 6–20 Borg scale. (Pageaux 2016). Each participant responded to the question “How hard is it for you to drive your legs and arms, and how heavy is your breathing?” on a scale of 6 (no exertion at all) to 20 (maximal exertion) (Fig. 1) after the 20-km cycling TT. Moreover, dyspnea was quantified using a 0–10 scale, with 0 indicating no dyspnea and 10 indicating maximal dyspnea. Similarly, pain was quantified using a 0–10 scale, where 0 represented no pain and 10 represented maximal pain. During the three days of testing, carbohydrate-supplemented athletes consumed the same brand, formulation, and quantity of carbohydrate. Hydration was allowed *ad libitum* on all testing days.

### Statistical analyses

The sample size was calculated using the total time for the sprint triathlon as the primary outcome variable. Drawing upon previously published literature (Vivan et al. 2024), and considering an alpha level of 0.05, a statistical power of 0.80, and an effect size of 0.41, the minimum recommended sample size was estimated be 6 participants of each sex. Anticipating potential attrition throughout the data collection period, we opted to commence the study with a larger participant cohort of 12 men and 10 women.

The descriptive data (mean and standard deviation) were presented. Effect sizes, and effect size confidence intervals were also calculated and presented. Statistical analysis was performed using a repeated-measures analysis of variance, with the three intensities as the within-subject factor, and sex (male and female) as the between-subject factor. Partial eta squared was used as a surrogate for effect size. Data were tested for normality using the Shapiro–Wilk test. Sphericity was assessed by the Mauchly test; when the sphericity assumption was violated, the Greenhouse-Geisser correction was applied. The measures of effect size for the differences between the groups were determined by calculating the mean difference between the two groups and dividing the result by the pooled standard deviation. The magnitude of any change was judged based on the following criteria: d < 0.2 was considered to have no effect, 0.2 ≤ d <0.5 was considered a small effect size, 0.5 ≤ d <0.8 represented a medium effect size, 0.8 ≤ d < 1.3 was regarded a large effect size, and d ≥ 1.3 was regarded as a very large effect size (Sullivan and Feinn 2012). The significance level adopted for all analyses was p ≤ 0.05. In case of significant main effects or interactions, post hoc tests were performed with Sidak’s adjustment for multiple comparisons. The analyses tested for the main effects of intensity, sex, and the time × sex interaction. All statistical procedures were performed using the software SPSS Statistics version 28.0 (IBM Corp., USA).

## Results

### Swimming

The CV for male athletes was 0.96 ± 0.11 m/s. In the trial the men swam slower, their average velocity was 0.92 ± 0.11 m/s. In the trial they swam at CV, average velocity was 0.96 ± 0.12 m/s, and in the trial, they swam faster than their CV, the average velocity was 0.99 ± 0.12 m/s. Thus, the intended between-day difference in average swimming speed was approximately 3%.

The CV for female athletes was 0.82 ± 0.11 m/s. In the trial the women swam slower than their CV, the average velocity was 0.80 ± 0.11 m/s. In the trial they swam at intermediate velocity, the average velocity was 0.83 ± 0.12 m/s, and in the trial, they swam faster than their CV, the average velocity was 0.86 ± 0.12 m/s. The average between-day speed difference was roughly 3%.

We thank the reviewer for this observation. The swimming intensities performed on days 3, 4, and 5 were randomized. For the purposes of data analysis, we designated the lower-, intermediate-, and higher-intensity sessions as 1, 2, and 3, respectively. Thus, the numbers 1, 2, and 3 refer to the intensity levels performed rather than to the chronological testing days. The text has been revised to clarify this point. For the male group, intensity had a significant effect on blood lactate level measured at the end of swimming on each day, being significantly higher on day 3 than on day 2 (*p* = 0.001) and day 1 (*p* < 0.001). Additionally, the lactate level on day 2 was significantly higher than on day 1 (*p* = 0.002). There was no significant difference in lactate concentration between the days for the female group. Furthermore, sex had a significant effect on blood lactate level. Men presented significantly higher average blood lactate levels than women on days 2 (*p* = 0.024) and 3 (*p* = 0.011). There was no significant interaction between sex and day in influencing blood lactate level [F(2,28) = 2.870; *p* = 0.073] (Table 2).

**Table 2:** Swimming data for men and women

Swimming	Sex	Day 1	Day 2	Day 3	ANOVA	F	*p* value	Partial eta square	Power
Lactate (mmol/L)	Men (n = 10)	4.7 ± 1,0*#	6.8 ± 1.8#&	8.5 ± 1.6&	Intesity group	27.460	< 0.001	0.662	1.000
	Women (n=6)	3.7 ± 1.3	4.6 ± 1.4	5.6 ± 2.4	Sex group	8.651	0.011	0.382	0.781
					Interaction	2.870	0.073	0.170	0.517
RPE	Men (n = 10)	11 ± 2*#	14 ± 2#	17 ± 3	Intesity group	26.390	< 0.001	0.653	1.000
	Women (n = 6)	11 ± 2*#	14 ± 2	16 ± 3	Sex group	0.616	0.446	0.042	0.113
					Interaction	0.515	0.603	0.036	0.126
HR (bpm)	Men (n = 10)	136 ± 14	148 ± 17	150 ± 15	Intesity group	4.116	0.027	0.227	0.681
	Women (n = 6)	132 ± 19	137 ± 15	146 ± 14	Sex group	1.514	0.239	0.098	0.209
					Interaction	0.439	0.649	0.030	0.114
Pain	Men (n = 10)	3 ± 2*#	4 ± 2&	6 ± 1&	Intesity group	9.957	0.001	0.411	0.971
	Women (n = 6)	2 ± 1	2 ± 2	4 ± 2	Sex group	6.263	0.025	0.309	0.644
					Interaction	2.123	0.139	0.132	0.398
Dyspnea	Men (n = 10)	3 ± 1*#&	5 ± 2#	7 ± 2	Intesity group	30.535	< 0.001	0.686	1.000
	Women (n = 6)	2 ± 1*#	4 ± 1	5 ± 2	Sex group	5.433	0.035	0.280	0.583
					Interaction	0.260	0.773	0.018	0.087

Data are presented as mean ± standard deviation. Confidence Interval = 95%

*HR* Heart rate, *RPE* Rating of perceived exertion

^*^p < 0.05 (different from Day 2), #*p* < 0.05 (different from Day 3); &*p* < 0.05 (different from women at the same moment)

The RPE in men exhibited the same behavioral pattern about intensity effect as blood lactate level, with significantly higher levels on day 3 than on day 2 (*p* = 0.019) and day 1 (*p* < 0.001). Additionally, the average RPE on day 2 was significantly higher than on day 1 (*p* = 0.003). For women, the RPE on day 1 was significantly lower than on day 2 (*p* = 0.037) and day 3 (*p* = 0.011), with no significant difference observed between days 2 and 3. Furthermore, there was no significant difference in RPE between sexes [F(1,14) = 0.616; *p* = 0.446], nor interaction between sex and day [F(2,28) = 0.515; *p* = 0.603] (Table 2). For HR, a significant effect of intensity was observed [F(2,28) = 4.116; *p* = 0.027], but there was no significant sex effect [F(1,14) = 1.514; *p* = 0.239], and no interaction effect [F(2,28) = 0.439; *p* = 0.649] (Table 2).

Regarding pain, there was a significant effect of intensity and the perception of pain for men was significantly lower on day 1 than on days 2 (*p* = 0.025) and 3 (*p* = 0.004); there was no significant difference (*p* = 0.490) between days 2 and 3. For women, there was no significant difference between the days. There was a significant effect of sex on pain perception, with men presenting significantly higher values on the pain scale than women on days 2 (*p* = 0.015) and 3 (*p* = 0.024). There was no interaction effect [F(2,28) = 2.123; *p* = 0.139] (Table 2). For dyspnea, there was a significant effect of intensity; the perception for men was significantly lower on day 1 than on days 2 (*p* < 0.001) and 3 (*p* < 0.001). Additionally, perception of dyspnea on day 2 was significantly lower than on day 3 (*p* = 0.039). For women, the perception of dyspnea was significantly lower on day 1 than on days 2 (*p* = 0.002) and 3 (*p* = 0.002), with no significant difference (*p* = 0.909) between days 2 and 3. Men had a significantly higher average dyspnea value than women on day 1 (*p* = 0.037). There was no interaction effect of days and sex [F(2,28) = 0.260; *p* = 0.773] (Table 2).

### Cycling

**Fig. 2:**
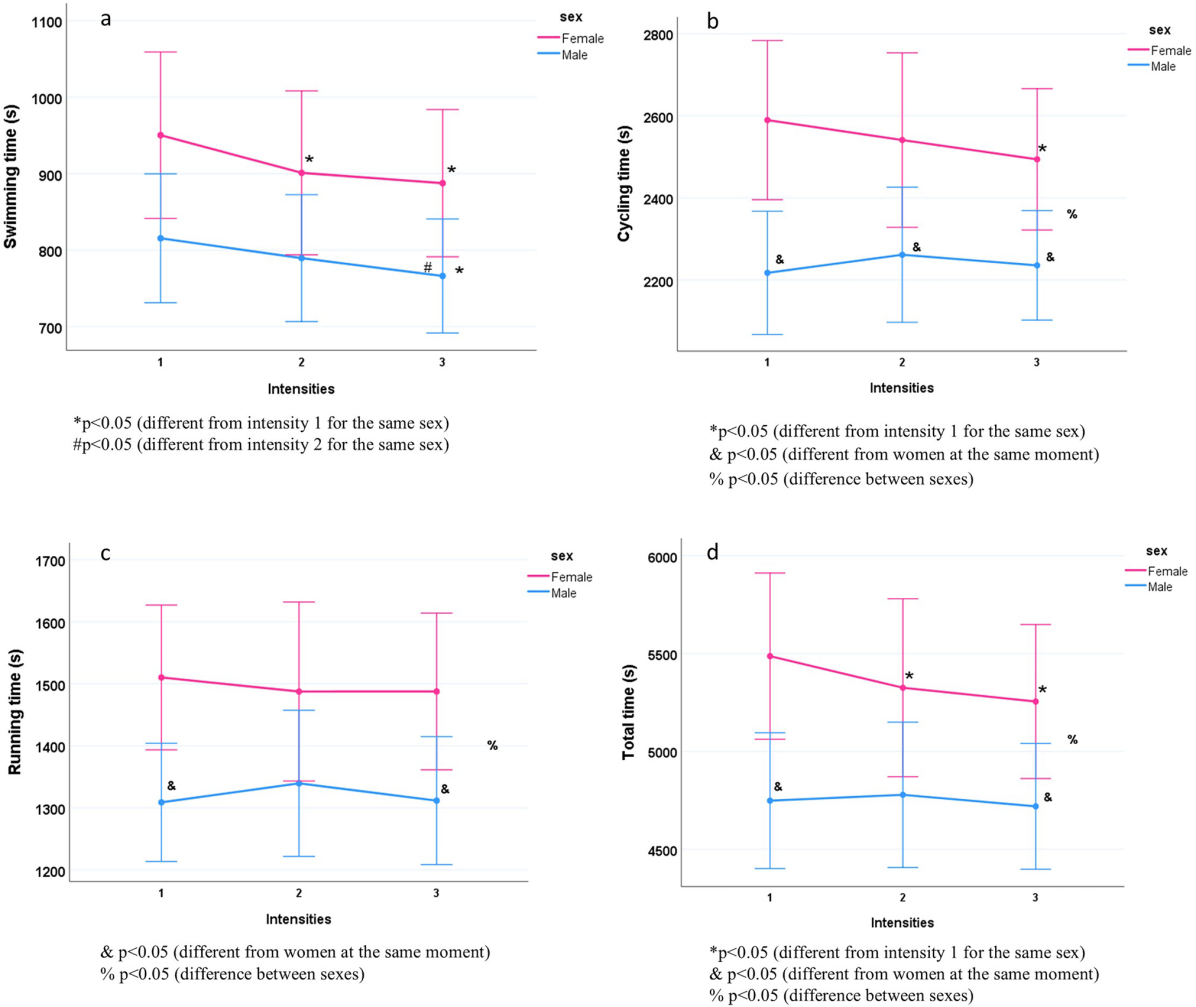
Time and intensity in different modalities and total time in Triathlon Sprint

For men, there were no significant differences in time to cycle 20 km between days. However, for women, the cycling time on the day of lowest intensity (day 1) was significantly higher than that on the day of higher intensity (day 3) (*p* = 0.023). Between days 1 and 2 (*p* = 0.421), and between days 2 and 3 (*p* = 0.427), there were no significant differences. Additionally, men presented significantly lower times compared to women on days 1 (*p* = 0.006), 2 (*p* = 0.043), 3 (*p* = 0.023). There was a significant effect of the interaction between day and sex [F(2,28) = 4.424; *p* = 0.021] (Fig. 2) on time to cycle 20 km.

There was no significant effect of sex [F(1,14) = 1.089; *p* = 0.314], day [F(2,28) = 0.909; *p* = 0.415] nor the interaction between sex and day [F(2,28) = 0.146; *p* = 0.865] on blood lactate level after cycling.

Sex did not significantly influence [F(1,14) = 1.844; *p* = 0.196] RPE after cycling, but day had a significant [F(2,28) = 3.943; *p* = 0.049] influence on it. There was no significant interaction between sex and day [F(2,28) = 1.431; *p* = 0.257] in influencing RPE after cycling.

For HR, there was no significant effect of sex [F(1,14) = 0.061; *p* = 0.809] and day [F(2,28) = 0.915; *p* = 0.412]. Sex and day did not have significant interaction effects [F(2,28) = 0.040; *p* = 0.961] on HR after cycling.

For pain perception, there was also no significant effect of sex [F(1,14) = 2.974; *p* = 0.107], but there was a significant effect of day [F(2,28) = 3.374; *p* = 0.049]. There was no interaction effects of sex and day [F(2,28) = 0.541; *p* = 0.588] on pain perception after cycling.

Finally, for dyspnea there was also no effect of sex [F(1,14) = 0.101; *p* = 0.755], day [F(2,28) = 1.012; *p* = 0.376], nor interaction between sex and day [F(2,28) = 0.544; *p* = 0.586] (Table 3).

**Table 3:** Cycling data for men and women

Cycling	Sex	Day 1	Day 2	Day3	ANOVA	F	*p* value	Partial eta square	Power
Lactate (mmol/L)	Men (n = 10)	4.0 ± 1.9	4.2 ± 17	4.4 ± 1.3	Intesity group	0.909	0.415	0.061	0.191
	Women (n = 6)	3.2 ± 0.6	3.6 ± 1.0	3.8 ± 1.0	Sex group	1.089	0.314	0.072	0.164
					Interaction	0.146	0.865	0.010	0.070
RPE	Men (n = 10)	16 ± 2	16 ± 2	16 ± 2	Intesity group	3.943	0.049	0.220	0.550
	Women (n = 6)	14 ± 2	15 ± 1	15 ± 1	Sex group	1.844	0.196	0.116	0.244
					Interaction	1.431	0.257	0.093	0.235
HR (bpm)	Men (n = 10)	149 ± 20	151 ± 15	152 ± 20	Intesity group	0.915	0.412	0.061	0.192
	Women (n = 6)	147 ± 14	149 ± 10	151 ± 9	Sex group	0.061	0.809	0.004	0.056
					Interaction	0.040	0.961	0.003	0.055
Pain	Men (n = 10)	6 ± 2	6 ± 1	7 ± 1	Intesity group	3.374	0.049	0.194	0.588
	Women (n = 6)	4 ± 3	4 ± 3	5 ± 2	Sex group	2.974	0.107	0.175	0.362
					Interaction	0.541	0.588	0.037	0.130
Dyspnea	Men (n = 10)	4 ± 2	4 ± 2	4 ± 1	Intesity group	1.012	0.376	0.067	0.208
	Women (n = 6)	4 ± 1	4 ± 1	4 ± 2	Sex group	0.101	0.755	0.007	0.060
					Interaction	0.544	0.586	0.037	0.131

Data are presented as mean ± standard deviation. Confidence Interval = 95%

*HR* Heart rate, *RPE* Rating of perceived exertion

### Running

In the last stage of the triathlon, the time to complete a 5-km time trial did not significantly differ between days [F(2,26) = 0.474; *p* = 0.628), and there was no interaction effect [F(2,26) = 1.647; *p* = 0.212]. However, men recorded a significantly lower running time than women on days 1 (*p* = 0.013) and 3 (*p* = 0.037) (Fig. 2).

For all physiological variables analyzed after the running time trial, there was no significant difference in blood lactate level [F(2,28) = 0.309; *p* = 0.737], RPE [F(2,28) = 1.550; *p* = 0.231], HR [F(2,28) = 1.722; *p* = 0.198], pain [F(2,28) = 0.524; *p* = 0.504] and dyspnea [F(2,28) = 0.235; *p* = 0.793] between the days.

In addition, there was no difference between sexes in blood lactate level [F(1,14) = 2.565; *p* = 0.133], HR [F(1,14) = 0.149; *p* = 0.706], pain [F(1,14) = 1.045; *p* = 0.325], and dyspnea [F(1.14) = 2.893; *p* = 0.113]. For RPE, women presented significantly higher values than men on days 1 (*p* = 0.008) and 2 (*p* = 0.032). There was no significant interaction effect between sex and day on any of the variables (Table 4).

**Table 4:** Running data for men and women

Running	Sex	Day 1	Day 2	Day3	ANOVA	F	*p* value	Partial eta square	Power
Lactate (mmol/L)	Men (n = 9)	6.2 ± 2.3	5.9 ± 1.4	6.4 ± 1.7	Intesity group	0.309	0.737	0.023	0.094
	Women (n = 6)	7.3 ± 1.9	8.0 ± 1.3	7.4 ± 1.2	Sex group	2.565	0.133	0.165	0.317
					Interaction	1.879	0.173	0.126	0.355
RPE	Men (n = 9)	18 ± 2&	18 ± 1&	18 ± 2	Intesity group	1.550	0.231	0.107	0.299
	Women (n = 6)	20 ± 1	20 ± 1	20 ± 1	Sex group	6.613	0.023	0.337	0.662
					Interaction	1.550	0.231	0.107	0.299
HR (bpm)	Men (n = 9)	172 ± 9	173 ± 12	175 ± 7	Intesity group	1.722	0.198	0.117	0.328
	Women (n = 6)	166 ± 18	174 ± 12	177 ± 11	Sex group	0.149	0.706	0.011	0.065
					Interaction	0.593	0.560	0.044	0.138
Pain	Men (n = 9)	7 ± 2	8 ± 4	7 ± 2	Intesity group	0.524	0.504	0.039	0.107
	Women (n = 6)	6 ± 3	6 ± 3	7 ± 3	Sex group	1.045	0.325	0.074	0.158
					Interaction	2.194	0.158	0.144	0.299
Dyspnea	Men (n = 9)	7 ± 3	8 ± 1	7 ± 2	Intesity group	0.235	0.793	0.018	0.083
	Women (n = 6)	9 ± 2	8 ± 2	9 ± 1	Sex group	2.893	0.113	0.182	0.351
					Interaction	1.384	0.268	0.096	0.270

Data are presented as mean ± standard deviation. Confidence Interval = 95%

*HR* Heart rate, *RPE* Rating of perceived exertion

^&^*p* < 0.05 (different from women at the same moment

### Total time

For women, on the day when swimming was slower than the CV, the overall race time was higher than on the day when swimming was at CV (*p* = 0.041) and faster than the CV (*p* = 0.002). There was no significant difference in overall race time between the days when swimming was at CV and higher than CV. For men, there was no significant difference in the overall race time among the three different intensities. However, men had significantly shorter average overall race time than women when swimming was lower (*p* = 0.012) and higher (*p* = 0.040) than CV, and there was a significant effect of interaction between sex and day [F(2,26) = 5.996; *p* = 0.007) (Fig. 2)*.*

### Transition times

For the male group, the transition time between swimming and cycling (T1) for the lower-, intermediate-, and higher-intensity sessions were 299 ± 101 s, 297 ± 91 s, 301 ± 89 s, respectively. For the female group, the transition time between swimming and cycling (T1) for the lower-, intermediate-, and higher-intensity sessions were 332 ± 104 s, 256 ± 29 s, 272 ± 26 s, respectively.

For the male group, the transition time between cycling and running (T2) for the lower-, intermediate-, and higher-intensity sessions were 113 ± 28 s, 110 ± 18 s, 110 ± 23 s, respectively. For the female group, the transition time between swimming and cycling (T1) for the lower-, intermediate-, and higher-intensity sessions were 109 ± 10 s, 107 ± 19 s, 128 ± 54 s, respectively.

The transition time between swimming and cycling (T1) did not significantly differ between days [F(2,32) = 1.245; *p* = 0.302] or sexes [F(1,16) = 0.047; *p* = 0.831]. Similarly, the transition time between cycling and running (T2) did not differ significantly between days [F(2,30) = 0.476; *p* = 0.626] and the sexes [F(1,15) = 0.001; *p* = 0.991].

## Discussion

This study aimed to investigate the effect of swimming intensity on performance in subsequent cycling and running, and total race time for triathlon in well-trained female and male amateur triathletes. This study hypothesizes that intense swimming may impair subsequent cycling and running performance, with potential consequences for the overall race outcome.

The main results of this study were that (i) for men, swimming intensity did not have a significant effect on overall race time; (ii) for women, lower swimming intensity was associated with longer overall race time; (iii) swimming intensity had a significant effect on internal load variables, such as blood lactate level and RPE, and external load variables, such as swimming velocity for both sexes; (vi) swimming intensity did not significantly influence cycling and running performance for men, but the slowest swimming day generated a significantly lower cycling velocity for the women group; and (v) men performed better than women in swimming, cycling and running. Therefore, the findings generally did not confirm the study’s hypothesis.

In this study, all athletes swam at three different intensities according to their CV. It is worth noting that the protocol selected for the assessment of critical velocity (CV) was based on two distances, 200 m and 400 m, which has been previously tested and validated (Costa et al. 2009; Toubekis et al. 2011b). Interestingly, the swimming velocity over 400 m (v400m) has been shown to closely reflect maximal aerobic performance, with previous research indicating that maximal aerobic velocity corresponds to approximately 92% of v400m (Zacca et al. 2016). The results indicated that the three proposed swimming intensities produced different performances. On the most intense day, the time taken to swim 750 m was significantly shorter than on the lighter-intensity days, resulting in considerably greater physiological stress, as evidenced by higher blood lactate levels, heart rates, pain, dyspnea, and RPE levels for both female and male groups. These results confirm that each day generated a different physiological stress level to perform the subsequent cycling and running splits. The central question of this study was prompted by previous studies suggesting that significant exertion during swimming might lead to substantial physiological stress, impacting performance in subsequent cycling and running (Margaritis 1996; Bentley et al. 2002; Millet et al. 2003).

However, swimming intensity did not negatively impact performance in subsequent modalities, except for the male athletes. The lack of difference in overall race performance between the days, despite the different swimming intensities, might be attributed to the fact that the percentage contribution of swimming in a sprint triathlon is lower than that of cycling and running. Swimming typically accounts for 15%–20% of the total race time, whereas cycling contributes 50%–55%, and running constitutes 30%–35% (Figueiredo et al. 2016; Sousa et al. 2021). Moreover, the most important split time to overall performance in sprint triathlon is the cycling time, not the swimming time (Sousa et al. 2021). Barragan et al. (2020), who conducted a similar study, also reported no significant differences in the times for the cycling and running stages across swimming conditions; however, the overall triathlon performance was better when the athletes swam at a higher intensity. Possibly, the overall differences in performance were caused by more pronounced differences in the swimming intensity (approximately 10%) between days in that study, than in our study (about 3%). In contrast, in a similar study, Peeling et al. (2005) reported that swimming at the highest intensity—100% of the 750-m swim time trial—compromised cycling performance and the overall triathlon race time. A key difference that might have contributed to the discrepancies with our findings is that in Peeling et al. (2005), the highest swimming intensity was the maximum an athlete could achieve, whereas in our study the highest swimming intensity was 3% above VC, and in Barragan et al.’s study, it was 90% of the highest swimming intensity. Another difference between the studies that might have affected the results is that Peeling et al. (2005) used highly trained athletes as participants, whereas we and Barragan et al. (2020) assessed moderately trained athletes.

In contrast, female athletes presented different results. They had the worst cycling time and overall race performance on the day, with the least swimming intensity. Possibly, they had difficulty in increasing their effort intensity after an easy swim, leading to slower cycling than on other days. Although no clear evidence exists that starting at an excessively slow pace permanently diminishes neuromuscular activation, a state of diminished pre-activation has the potential to compromise performance (McGowan et al. 2015).This was evidenced by the blood lactate levels, which remained constant during the subsequent cycling after the easy swim.

Female athletes reached lower blood lactate levels, pain, and dyspnea after a swim than the male athletes. These lower levels experienced by female athletes could be associated with their higher maximal rates of fatty acid oxidation and carbohydrate preservation (Venables et al. 2005; Lundsgaard and Kiens 2014). The greater proportion of type I muscle fibers, higher oxidative capacity, and the greater muscle capillarization observed in women than in men might also explain the lower blood lactate levels among the female athletes (Bassett et al. 2020). Vivan et al. (2024) confirmed that male athletes utilize glycolytic metabolism more than female athletes, leading to higher blood lactate levels. Additionally, they have shown that higher cycling intensity may impair the subsequent running performance for male athletes. In contrast, for female athletes, cycling intensity has a different effect, suggesting that women perform the event more aerobically.

Differences between females and males in split times and overall race performance were within the expected range for sports; women’s performance was generally lower than that of men. However, female triathletes are progressively closing the performance gap with their male counterparts. The performance gap between men and women has been decreasing over the last three decades, varying between 12% and 18% depending on the distance and ability of the athlete (Lepers et al. 2013). Etter et al (2013) observed that, in the non-drafting Zurich Olympic Triathlon, the differences in 1.5-km swimming, 40-km cycling, 10-km running, and total event times were 18.5%, 15.5%, 18.5%, and 17.1%, respectively. Additionally, the number of women finishing an Ironman in under 9 hours has risen from just one in 1991 to 23 in 2017 (Lepers 2019). Possibly, this reduction in the performance gap between sexes can be attributed, at least in part, to increased female participation in sports since the 1990s, although they still represent a smaller portion of the total competitors, ranging from 25% to 40% in events (Lepers 2008; Lepers et al. 2013).

Differences between females and males observed in swimming were less pronounced in running, which in turn exhibited less pronounced differences than in cycling. These differences might be partly explained by physiological and morphological factors. Female athletes generally have 7%–12% more body fat than male athletes (Fleck 1983). As fat is more buoyant, women tend to be less affected by swimming than by cycling and running (Lepers 2019). Conversely, cycling performance relies on a combination of aerobic capacity and muscular power (Rønnestad and Mujika 2014), which is significantly lower among female athletes than male athletes; therefore, the sex differences in cycling are expected to be greater. Additionally, in cycling and running, performance depends primarily on conditioning, strength, and power, but swimming performance can be expressed as the ratio of available metabolic power (E′) to the energy cost of locomotion, which is highly dependent on technical skill. (Olbrecht 2011).

## Limitations and strengths of the study

This study had some limitations that should be considered. The sample was restricted to swimmers with similar competitive levels, which limits the generalizability of the results. Additionally, possible physiological confounding factors, such as the menstrual cycle in women, were not controlled, which could have influenced the response to effort on different days. Furthermore, the experiment was conducted in a swimming pool, under controlled conditions, which were different from those in a real competition. Moreover, the athletes did not use wetsuits for the protocol, which could have reduced fatigue during swimming (Quagliarotti et al. 2023). Furthermore, another potential limitation concerns the 10-min interval between the two maximal efforts used to determine CV (200 m and 400 m). Although heart rate, perceived exertion, and dyspnea were monitored to ensure participants had returned to baseline values prior to initiating the second test, complete recovery cannot be fully guaranteed, therefore the authors recommend interpreting the absolute CV values with caution. In this context, it is worth noting that the main purpose of calculating CV in the present study was to provide a reference parameter to guide swimming intensity, ensuring that each session was performed at a distinct effort level—an objective that was successfully achieved. A strength of the study was incorporating sex as a variable and analyzing its effect on performance responses, which had not been done in previous studies. This design allowed us to investigate sex differences in how swimming intensity affects triathlon performance.

## Practical application

The results suggest that men and women respond differently to variations in intensity, which is reflected in the modalities and total times during a sprint triathlon event. For women, starting the swimming segment at a very light pace has the potential to impair overall race performance; however, this does not appear to be the case for the male group. These findings underscore the importance of considering an athlete’s sex when customizing training prescriptions. Moreover, although the different swimming velocities performed on each day are modest in magnitude, they are highly significant. In swimming, such differences may correspond to distinct training intensity domains. Therefore, rigorous monitoring of swimming velocity by coaches is warranted.

## Conclusion

This study aimed to compare the impact of swimming intensity on partial and total times in a sprint triathlon event between men and women. The findings indicated that for men, swimming intensity had no impact on the overall triathlon time. Moreover, neither the cycling nor the running split times were significantly influenced by swimming intensity. Regarding women’s performance, the results show that on the day with the highest intensity swimming, cycling was also performed best, and running wasn’t impacted. Consequently, the day with the most intense swimming was also the day with the best overall triathlon time. In addition, men had lower total times than women at moderate and very intense intensities.

## Data Availability

Data supporting the study results can be provided followed by request sent to the corresponding author’s e-mail.

## Abbreviations

CV: Critical velocity
DXA: Dual-energy X-ray absorptiometry
VT: Ventilatory threshold
RCP: Respiratory compensation point
V̇O_2_max: Maximum oxygen consumption
MAP: Maximal aerobic power
HRmax: Maximal heart rate
HR: Heart rate
TT: Time trial
T1: First transition
T2: Second transition
RPE: Rating of perceived exertion
